# Intrauterine administration of platelet‐rich plasma improves embryo implantation by increasing the endometrial thickness in women with repeated implantation failure: A single‐arm self‐controlled trial

**DOI:** 10.1002/rmb2.12334

**Published:** 2020-06-25

**Authors:** Maki Kusumi, Tatsuji Ihana, Takako Kurosawa, Yasuo Ohashi, Osamu Tsutsumi

**Affiliations:** ^1^ Center for Human Reproduction and Gynecologic Endoscopy Sanno Hospital Tokyo Japan; ^2^ Department of Integrated Science and Engineering for Sustainable Society Chuo University Tokyo Japan; ^3^ Director of Sanno Hospital Tokyo Japan

**Keywords:** assisted reproductive technology, fertilization in vitro, hormone replacement therapy, intracytoplasmic sperm injection, platelet‐rich plasma

## Abstract

**Purpose:**

The purpose of this study was to investigate the effectiveness of intrauterine administration of platelet‐rich plasma (PRP) in frozen embryo transfer (FET) cycle in Japanese patients with a thin endometrium.

**Method:**

A prospective single‐arm self‐controlled trial was conducted in Japan. PRP administration was performed in 36 of the 39 eligible patients with a thin endometrium (≤7 mm). Hormone replacement therapy (HRT) with estrogen was performed for 2 menstrual cycles, and PRP was administrated on the 10th and 12th days of the second HRT cycle. The endometrial thickness was evaluated on transvaginal ultrasonography by two physicians at every visit, one an attending physician and the other a specialist physician blinded to the date and timing of the sonography. FET was performed during the second HRT cycle after PRP administration.

**Results:**

After PRP administration, the mean (SD) endometrial thickness on the 14th day was significantly increased by 1.27 mm (*P* < .001) and 0.72 mm (*P* = .001) on the basis of the unblinded and blinded measurements, respectively. Of the 36 patients, 32 (88.9%) underwent FET. The clinical pregnancy rate was 15.6%. No adverse events occurred.

**Conclusions:**

PRP therapy was safe and effective in increasing endometrial thickness improving possibly pregnancy rate.

## Graphical abstract

It is the first report to show that PRP improved endometrial thickness improving possible pregnancy rate in infertile patients from Japan. As a new approach, we have tried to exclude measurement bias by the attending physician, by measuring endometrial thickness independently by the other physician who is blind to the date and timing of sonography.

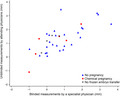

## 1 INTRODUCTION

According to the 2017 tabulation results of the assisted reproductive technology (ART) registry conducted by the Japan Society of Obstetrics and Gynecology, the number of ART cycles performed in Japan was the highest in the world at 448 210 cycles, and the number of births through ART was 56 617, accounting for 1 in 16.7 births. The mean patient age was 38.0 years, but most patients were treated with ART at the age of 40 years or more. Of all the embryo transfer cycles, 55 720 (22.3%) were fresh embryo transfer cycles and 194 415 (77.7%) were frozen embryo transfer (FET) cycles. The number of pregnancies after FET was 66 881 (pregnancy rate per FET, 34.4%), while the number of births was 47 807 (live birth rate per FET, 24.6%). Of all the FETs, 83.4% were single‐embryo transfers (SETs); 96.7%, singleton pregnancies; and 96.6%, singleton births.^1^ The pregnancy rate by FET in Japan is low as compared with those in other countries according to the International Committee Monitoring Assisted Reproductive Technologies (ICMART), partly because the targets of ART in Japan are older women, and preimplantation genetic testing for aneuploidy (PGT‐A) is currently not allowed by the Japan Society of Obstetrics and Gynecology.

Overall, the freeze‐all/FET strategy in ART contributes to the improvement of pregnancy rates. This is because controlled ovarian stimulation increases the number of oocytes retrieved and prevents ovarian hyperstimulation syndrome,^2^ enabling avoidance of the effect of a non‐physiological endocrine environment due to controlled ovarian stimulation on implantation.^3^ Moreover, for women aged ≥38 years, the predominance of FET is increasing especially with the improving pregnancy rates and decreasing abortion rates due to PGT‐A.^4^, ^5^

Improvement in the implantation environment has become a major focus area of ART because of the prevalence of recurrent implantation failure (RIF). RIF represents cases of women aged ≤40 years who do not become pregnant even after at least 3 embryo transfers (≥4 embryos).^6^ RIF has various causes, of which endometrial thinning (<7 mm; at the initiation of progestin therapy, during human chorionic gonadotropin administration [hCG], or at the time of embryo transfer) is an important factor for implantation failure.^7^, ^8^ Meta‐analysis showed that the population of thin endometrium (≤7 mm) was very small (only 2.4%) in IVF but the probability of clinical pregnancy in patients with thin endometrium ≤7 mm was significantly lower compared with patients with endometrial thickness >7 mm [23.3% versus 48.1%, OR 0.42 (95% CI 0.27‐0.67)].^8^ Administration of sildenafil for endometrial thinning may be an effective treatment but has not yet been verified in a randomized controlled trial (RCT). Therefore, new treatments are needed to improve endometrial thickness in patients with endometrial thinning.

Administration of platelet‐rich plasma (PRP) may be used as one of the methods to increase the implantation rate of fertilized eggs. PRP is easily prepared using plasma containing high concentrations of platelet‐derived growth factor and cytokines obtained from centrifugation of blood samples. The components of PRP include platelet‐derived growth factor, transforming growth factor beta, vascular endothelial growth factor, and epidermal growth factor. These components are considered to promote wound healing and tissue growth, and their use is expected to promote endometrial thickening in patients with a thin endometrium.^9^, ^10^ As stable implantation of fertilized eggs can be expected with thickening of the endometrium, PRP treatment is likely to contribute to increased implantation rates.

PRP has been applied in infertile patients with endometrial thinning. Chang et al^11^ confirmed that in 5 patients whose endometrial thickness was <7 mm (range, 5.9‐6.6 mm; mean, 6.3 mm), intrauterine injection of PRP in combination with hormone replacement therapy (HRT) thickened the endometrium to ≥7 mm (range, 7.0‐8.0 mm; mean, 7.5 mm) between 48 and 72 hours after PRP injection, and all 5 patients became pregnant by subsequent embryo transfer. Zadehmodarres et al^12^ administered HRT in combination with intrauterine PRP injection (twice on days 11‐12 and days 13‐14) to 10 patients in whom FET had been canceled because their endometrial thickness was ≤7 mm even after HRT. Thereafter, all the patients showed an endometrial thickness of ≥7 mm and underwent FET. The study showed that after FET, 5 patients became pregnant, including one whose pregnancy resulted in spontaneous abortion and 4 who maintained a normal pregnancy. Recent RCTs of PRP have shown that PRP improves endometrial thickness and increases implantation rates.^13^ PRP has also been applied in patients with RIF^14^ and may represent a potential therapy to optimize the preparation of the endometrium for successful implantation. We conducted a clinical study of PRP in patients with a thin endometrium (≤7 mm) who were scheduled to undergo FET with HRT to investigate changes in endometrial thickness in detail and pregnancy outcomes.

## 2 MATERIALS AND METHOD

### 2.1 Study design and patients

This study was a prospective single‐arm self‐controlled trial conducted in Japan. This study was conducted from February 2018 through January 2019 at Sanno Hospital, Center for Human Reproduction and Gynecologic Endoscopy. Recruitment of the study participants and follow‐up survey was conducted at 7 fertility clinics in Japan. The objective of this study was to evaluate the efficacy and safety of PRP intrauterine infusion on endometrial thickness in women undergoing infertility treatments. In addition, we also performed a preliminary examination of the implantation rate. Women who had received ART and were going to receive FET with HRT were enrolled in this study. The inclusion criteria were age of 20‐50 years, oocyte retrieval performed at age 42 years or younger, and endometrial thickness of ≤7 mm. The exclusion criteria were hepatic disorders, hemoglobin level of <11 g/dL, platelet count of <150 000/mm^3^, use of anticoagulants, and pregnancy. All the patients provided written informed consent for participation in this trial. The study was conducted in accordance with the principles of the Declaration of Helsinki and the Act on the Safety of Regenerative Medicine.^15^ This study was approved by the Certified Committee for Regenerative Medicine (clinical study No. PB3170046) and by Sanno Hospital Ethics committees (approval No. 17‐S‐16) and submitted to the Ministry of Health, Labour and Welfare in Japan.

For HRT with estrogen, transdermal estradiol or oral estrogen tablets were used with a stable or step‐up regime from day2 or day3 of period, twice on each patients. Endometrial thicknesses were measured on transvaginal ultrasonography on the first through the third day from menstruation and the 10th, 12th (if available), and 14th day of the first and second HRT cycles. The endometrial thicknesses on the 14th day of the second HRT cycle were compared with those on the 14th day of the first HRT cycle. Hormonal measurements and PRP intrauterine infusion were both performed on the 10th and 12th day of the second HRT cycle. On the 14th day of the second HRT cycle, a clinical laboratory test was performed to test the adverse effect. Subsequently, vaginal progesterone suppository therapy was started. FET was performed on the 19th day or the appropriate date of the second HRT cycle. A pregnancy test that detects hCG level was performed 2 weeks after FET. Implantation (presence of a gestational sac [GS]) was assessed according to the routine procedure for evaluating embryo transfer. Data regarding the number of births were obtained after 1 year.

PRP was prepared from autologous blood as follows: Peripheral blood was drawn from the forearm using vacuum blood collection tubes (Acti‐PRP tube, Aeon International Inc, Taipei, Taiwan). Two tubes were used for collecting 20 mL (10 mL for each tube) of blood. The blood was centrifuged at 2000 *g* for 6 minutes, and a total of 1 mL (0.5 mL for each tube) of PRP were obtained. The entire PRP volume (1 mL) was infused into the uterine cavity as soon as possible with ET catheter (Kitazato Medical Co., Ltd.) under transvaginal ultrasound guidance without clamp.

### 2.2 Endpoints

The primary endpoint of the study was endometrial thickness. Three transvaginal ultrasonography images of the endometrium were taken for each patient, and the average of the three measurements from the three images, measured by two physicians, one an attending physician and the other a specialist physician blinded to the date and time of the transvaginal sonography, was recorded. The increments in thicknesses from the 10th to the 14th day of the second HRT cycle during with the PRP injections were compared with those of the first HRT cycle without PRP. The secondary endpoints were safety (adverse events) and patient pregnancy rate.

### 2.3 Statistical analysis

Analyses of the safety of PRP were performed in all eligible patients, while the analyses of efficacy were performed in the patients who received PRP injections on the 10th and 12th days of the second HRT cycle. A descriptive analysis of the baseline patient characteristics was performed using the mean, SD, for continuous variables and percentage for categorical variables.

For the analysis of endometrial thickness, which was the primary endpoint, the data on both the unblinded measurements by the attending physicians and the blinded measurements by the specialist physician were used. To investigate the correlation between the unblinded and blinded measurements, the Pearson correlation coefficient and its 95% confidence interval (CI) were calculated. Changes in endometrial thickness were calculated as “thickness on the 14th day—thickness on the 10th day of the second HRT cycle.” Moreover, the following changes in endometrial thickness were calculated and summarized using repeated‐measures analysis of variance as follows: “(thickness on the 14th day—thickness on the 10th day of the second HRT cycle)—(thickness on the 14th day—thickness on the 10th day of the first HRT cycle)” and “thickness on the 14th day of the second HRT cycle—thickness on the 14th day of the first HRT cycle.” For the analysis of the secondary endpoints, the number of adverse events, pregnancy rate, and implantation rate with its 95% CI was calculated.

Previous studies showed that the PRP intrauterine infusion increased endometrial thicknesses by a mean (SD) of 1.30 (0.21) mm^11^ and 2.45 (0.52) mm.^12^ In a Student *t* test, 5 patients were necessary for achieving 90% power under a one‐sided type 1 error of 2.5%, under the assumption that the intraindividual SD was 0.25‐0.50 mm and the effect to be detected was 1.2‐2.0 mm. We planned to enroll 30‐40 patients to perform exploratory analyses of the effect of PRP to consider each patient's background factors. Statistical analyses were performed with SAS version 9.4 (SAS Institute). Two‐sided *P* values (significance level of 0.05) were used in all the analyses.

## 3 RESULTS

### 3.1 Patients

Forty patients were enrolled in the study, and 1 patient was judged to be ineligible before starting HRT; thus, 39 eligible patients were included in the analysis set for safety. Among the 39 eligible patients, 3 withdrew from the study before PRP injection and 36 were included in the analysis set for efficacy.

The patients’ characteristics are shown in Table 1. The mean (SD) ages of the patients in the efficacy analysis set and at the time of embryo cryopreservation for transfer were 39.7 (3.2) and 38.1 (2.9) years, respectively. The mean (SD) maximum endometrial thickness was 5.86 (0.95) mm during the previous canceled FET cycle. Of the 36 patients included in the efficacy analysis, 22 (61.1%) had a history of pregnancies and 8 (22.2%) were parous women (Table S1). The most common causes of infertility included uterine myoma, endometrial polyps, and Asherman's syndrome (Table S2). All the patients in the efficacy analysis set had previously received multiple failed FET cycles as fertility treatment, with a mean (SD) number of cycles of 5.4 (5.4). In the efficacy analysis set, 30 patients (83.3%) had previously undergone surgeries, most commonly including hysteroscopic surgery (polyps, myomas), adnexal surgery, D&C, and myomectomy (Table S3).

**Table 1:** Patients’ characteristics

	All eligible patients (Safety analysis set)	Efficacy analysis set
	n = 39	n = 36
Age (years)		
Mean (SD)	39.7 (3.1)	39.7 (3.2)
Patient age (years) at the time of embryo cryopreservation		
BMI		
Mean (SD)	38.1 (2.9)	38.1 (2.9)
Mean (SD)	21.24 (2.11)	21.19 (1.90)
Endometrial thickness (mm)		
Mean (SD)	5.82 (0.98)	5.86 (0.95)
Infertility type^a^		
Primary	15 (38.5%)	14 (38.9%)
Secondary	24 (61.5%)	22 (61.1%)
Parity^a^		
Nulliparous	31 (79.5%)	28 (77.8%)
Parous	8 (20.5%)	8 (22.2%)
History of surgeries		
n (%)	33 (84.6%)	30 (83.3%)
Previous falied FET cycles		
Mean (SD)	5.7 (4.0)	5.6 (4.0)
Number of embryos transferred per cycle		
Mean (SD)	1.2 (0.4)	1.2 (0.3)

Abbreviations: BMI, body mass index; FET, frozen embryo transfer.

^a^ Details are shown in Table S1.

### 3.2 Efficacy and safety

Endometrial thickness increased during the second HRT cycle, and the mean (SD) increments in endometrial thickness between the 10th and 14th day of the second HRT cycle after PRP administration was 1.40 (0.84) mm on the basis of the unblinded measurements by the attending physicians and 0.50 (0.87) mm on the basis of the blinded measurements by the specialist physician (Table 2). The correlation coefficient between the unblinded and blinded measurements was 0.885 (95% CI, 0.854‐0.909) (Figure S1）.

**Table 2:** Endometrial thickness (in millimeters) in the HRT cycle with or without PRP

Measurement	HRT cycle	10th day	14th day	Increments from 10th to 14th
Unblinded	1st cycle (without PRP)	5.87 (0.89)	5.98 (0.86)	0.11 (0.81)
	2nd cycle (with PRP)	5.85 (1.03)	7.25 (1.11)	1.40 (0.84)
	Changes from the 1st to the 2nd cycle	‐0.02 (0.93)	1.27 (0.96) (*P* < .001)	1.29 (1.06) (*P* < .001)
Blinded	1st cycle (without PRP)	5.77 (1.14)	6.04 (0.93)	0.27 (0.99)
	2nd cycle (with PRP)	6.26 (1.06)	6.76 (1.24)	0.50 (0.87)
	Changes from the 1st to the 2nd cycle	0.49 (1.18)	0.72 (1.09) (*P < *.001)	0.23 (1.18) (*P* = .466)

Note

Values are reported as mean (SD) unless otherwise noted.

Abbreviations: HRT, hormone replacement therapy; PRP, platelet‐rich plasma.

**Figure 1:**
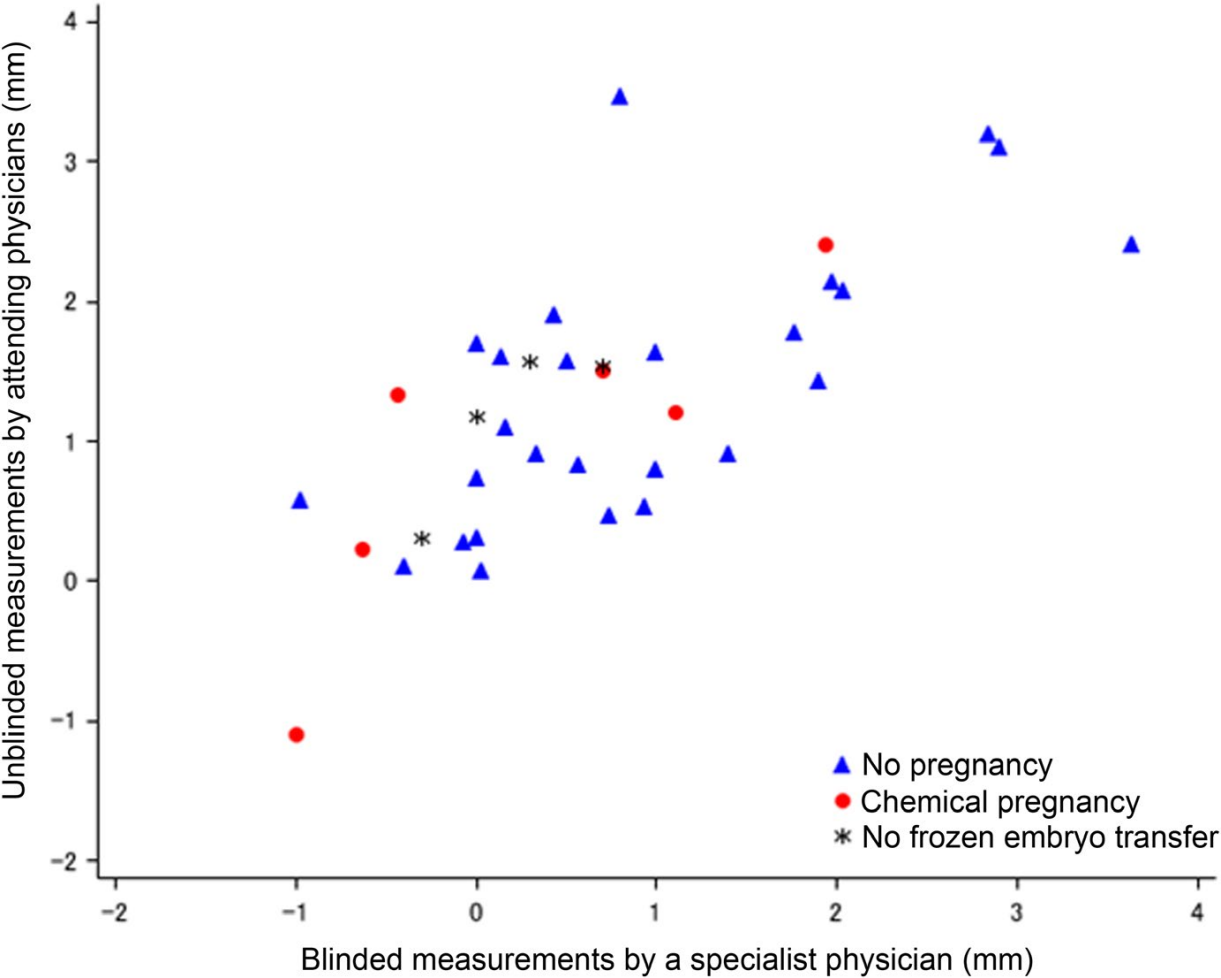
The scatter plot for the mean difference in endometrial thickness on the 14th day between the first Hormone replacement therapy (HRT) cycle without platelet‐rich plasma (PRP) and the second HRT cycle with PRP between the unblinded and blinded measurements, showing chemical pregnancy outcome. The increments in endometrial thickness caused by PRP administration are diverse. PRP administration increased endometrial thickness, but some patients were unresponsive to PRP therapy. The chemical pregnancy outcome was not associated with the increment in endometrial thickness

Changes in the endometrium thickness after PRP administration were investigated using an analysis of variance for longitudinal data. The endometrial thickness from the 10th to 14th day of the second HRT cycle increased by 1.29 mm (*P* < .001) on the basis of the unblinded measurements and by 0.23 mm (*P* = .466) on the basis of the blinded measurements, as compared with those in the first HRT cycle. The endometrial thickness on 14th day of the second HRT cycle was increased by 1.27 mm (*P* < .001) and 0.72 mm (*P* = .001) on the basis of the unblinded and blinded measurements, respectively, as compared with those in the first HRT cycle (Table 2). The scatter plot data of the mean difference in endometrial thickness showed a peculiar subgroup of patients with increment or almost no difference in endometrial thickness after PRP (Figure 1). Exploratory analyses for the effect of PRP considering the patients’ background factors were not performed because the number of patients in the subgroup was too small.

Regarding pregnancy rate, which was a secondary endpoint, 32 (88.9%) of the 36 patients underwent FET, whose mean (SD) endometrial thickness was 7.13 (0.89) mm. The mean (SD) number of transferred embryos was 1.1 (0.3) because only 4 of the 32 patients underwent double‐embryo transfer. After FET, the implantation rate (presence of a GS) was 13.9% (5/36 per transferred embryo; 95% CI, 4.7%‐29.5%); chemical pregnancy rate (hCG positive), 18.8% (6/32 per cycle; 95% CI, 7.2%‐36.4%); and clinical pregnancy rate (fetal heart beat or GS), 15.6% (5/32 patients; 95% CI, 5.3%‐32.8%). Regarding safety, no adverse events occurred in this study. The final outcomes of the clinical pregnancies in 5 patients were successful childbirth in 3 patients, spontaneous abortion in 1 patient, and unknown (lost to follow‐up) in 1 patient.

## 4 DISCUSSION

The endometrium plays a critical role in embryo implantation. The endometrium is a specific tissue that repeatedly falls and regenerates during each menstrual cycle, and appropriate endometrial thickness is required for implantation.^16^ All study patients (n = 39) had experienced canceled embryo transfer cycles because their endometrium showed no response to hormone therapy due to various reasons.

In this study, intrauterine PRP injection (on the 10th and 12th days from menstruation) significantly increased endometrial thickness. However, in the previous RCT that investigated the effect of PRP on endometrial thickness, the control group also showed an increase in the endometrial thickness,^13^ indicating a bias in measurements by the individual attending physicians. This study was designed as a self‐controlled study to compare endometrial thickness in the presence or absence of PRP administration in two identical hormone cycles. As a new approach, we excluded bias by measuring three images of the endometrium taken mostly by the same attending physician via transvaginal ultrasonography and by remeasuring the three transvaginal ultrasonography images by the other physician after blinding of the cycles and dates when the images were taken. The correlation between the unblinded and blinded measurements was high, which confirmed the reliability of measurements showed the efficacy of the PRP therapy in increasing endometrial thickness.

This study suggests that PRP administration may promote endometrial regeneration and showed that a clinical pregnancy was established after FET (number of transferred embryos: 1 ± 0.3) in 15.6% of the highly refractory infertile population with recurrent implantation failure in the prior therapy as shown in Table 1, that is, number of average failed cycle; 5.7. The absolute pregnancy rates in our study were lower than those in other reports, which may be due to the following reasons: (a) Among the 32 patients who underwent FET, 23 (71.9%) and 9 (28.1%) underwent blastocyst‐ and cleavage‐stage embryo transfers, respectively; the blastocyst‐stage transfer rate was relatively low in this study. (b) Single embryo transfer (SET) is mostly frequently used in Japan^17^ to prevent multiple pregnancies. Therefore, 87.5% (28/32) of our study patients underwent SET. (c) The mean age of the study patients was high (age at the time of oocyte collection: 38.7 ± 3.2 years). (d) In Japan, PGT‐A is not available because it is currently prohibited by the Japan Society of Obstetrics and Gynecology. (e) Patients with donor oocytes were not included in this study.

In other studies, embryo transfer was canceled when the endometrial thickness was <7 mm, and the cancellation rates of embryo transfer were 19.05% and 17.5%, respectively.^13^, ^18^ However, because the appropriate endometrial thickness for embryo transfer was not specified in this study, embryo transfer was canceled in 2 patients who presented with disease onset and in another 2 patients with a thin endometrium; thus, the embryo transfer cancellation rate due to endometrial thinning was extremely low (5.6%, 2/36 patients) as compared with that in the other studies. In fact, in some patients, the endometrium was unresponsive to the PRP treatment (Figure 1). Although the sample size of this study was small, 3 patients with a ≤7‐mm endometrium became pregnant after PRP administration. This finding indicates that PRP may improve the quality of the endometrium in addition to its thickness.

Most (83.3%) of the patients in this study had a history of surgery, of whom some had undergone multiple uterine surgeries, particularly D&C, total curettage, and hysteroscopic surgery. D&C leads to thinning of the endometrium.^19^ Only patients who presented with endometrial thinning several years after surgery were included in this study; thus, whether early postoperative intervention with PRP injection would be an option to regenerate the endometrium will need to be investigated. Comorbidities and a history of previous diseases resulting in surgeries cause decreased pregnancy rates depending on aging. It must be made known that such surgeries may reduce endometrium thickness. PRP therapy is also expected to be one of the treatment methods for endometrial regeneration.

Fertility treatment using PRP has been reported since 2015, and case reports have described cases of improved endometrial thickness and successful pregnancy/childbirth.^11^ Autologous PRP requires no culture and is characterized by ease of preparation and high safety. No adverse events were observed in this study. Although few basic studies have been conducted to investigate the effect of PRP on the endometrium, PRP therapy has been suggested to contribute to endometrial regeneration, increase blood flow in the uterus, and alleviate endometritis, which is currently considered a major factor of RIF. Moreover, PRP therapy has attracted attention as an effective therapy for RIF.^14^, ^20^, ^21^ Future research issues include investigation of the therapeutic effects of PRP on factors not limited to endometrial thickness, and the precise molecular mechanism of the effect of PRP must be elucidated.

## CONFLICT OF INTEREST

Maki Kusumi, Tatsuji Ihana, Takako Kurosawa, Yasuo Ohashi, and Osamu Tsutsumi declare that they have no conflict of interest.

## HUMAN RIGHTS STATEMENTS AND INFORMED CONSENT

All procedures followed were in accordance with the ethical standards of the responsible committee on human experimentation (institutional and national) and with the Helsinki Declaration of 1964 and its later amendments. Informed consent was obtained from all patients for being included in the study.

## APPROVAL BY ETHICS COMMITTEE

The protocol has been approved by a suitably constituted Ethics Committee.

Clinical Trial Registry: The trial registration number is UMIN000030493.

## DATA SHARING AND DATA ACCESSIBILITY

Not applicable.

## Supporting information

Fig S1

Table S1‐S3
