# Supplementary figures and images for: Intrauterine administration of platelet‐rich plasma improves embryo implantation by increasing the endometrial thickness in women with repeated implantation failure: A single‐arm self‐controlled trial
Source: Reprod Med Biol. 2020 Jun 25;19(4):350–6. doi: 10.1002/rmb2.12334 (PMC7542012; doi:10.1002/rmb2.12334)

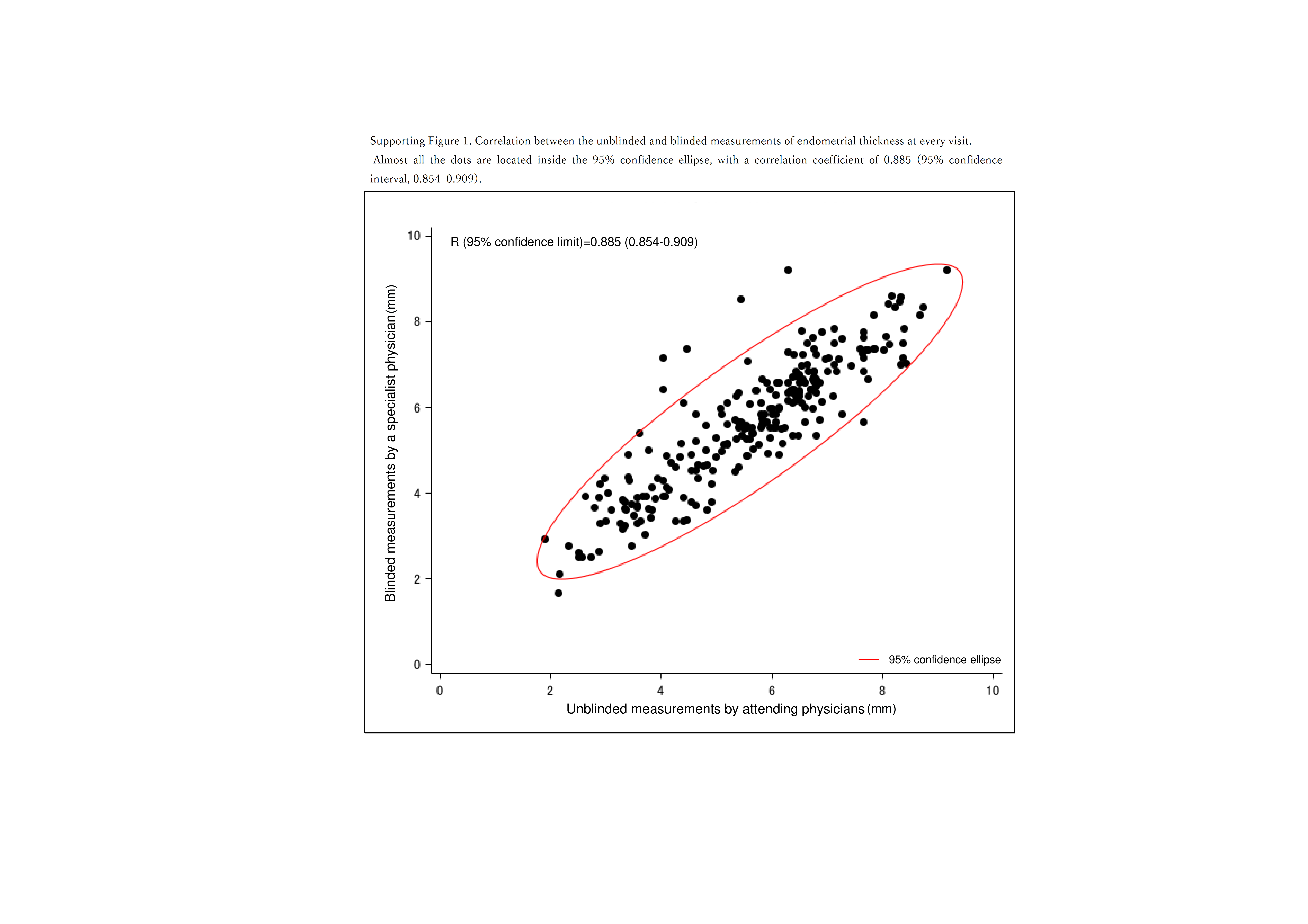

Supplement: Supplementary file 1 — Fig S1 [file RMB2-19-350-s001.tif]
