# Supplementary material for: Intrauterine administration of platelet‐rich plasma improves embryo implantation by increasing the endometrial thickness in women with repeated implantation failure: A single‐arm self‐controlled trial
Source: Reprod Med Biol. 2020 Jun 25;19(4):350–6. doi: 10.1002/rmb2.12334 (PMC7542012; doi:10.1002/rmb2.12334)
Supplement: Supplementary file 2 — Table S1‐S3 [file RMB2-19-350-s002.docx]

Supporting Table 1. Previous pregnancies

|  |  |  | All eligible patients  (Safety analysis set) | Efficacy analysis set |
| --- | --- | --- | --- | --- |
|  |  |  | n = 39 | n = 36 |
| Primary infertility | |  | 15 (38.5%) | 14 (38.9%) |
| Secondary infertility | |  | 24 (61.5%) | 22 (61.1%) |
| Spontaneous pregnancy | Term birth | Once | 2 (5.1%) | 2 (5.6%) |
| Abortion | Once | 5 (12.8%) | 5 (13.9%) |  |
|  | Twice | 4 (10.3%) | 3 (8.3%) |  |
|  | 4 times | 1 (2.6%) | 1 (2.8%) |  |
|  | 5 times | 1 (2.6%) | 1 (2.8%) |  |
| Fertility awareness methods | Term birth | Once | 1 (2.6%) | 1 (2.8%) |
| IUI | Term birth | Once | 1 (2.6%) | 1 (2.8%) |
| Abortion | Once | 3 (7.7%) | 3 (8.3%) |  |
| ART | Term birth | Once | 3 (7.7%) | 3 (8.3%) |
|  |  | 3 times | 1 (2.6%) | 1 (2.8%) |
| Stillbirth | Once | 1 (2.6%) | 1 (2.8%) |  |
| Abortion | Once | 10 (25.6%) | 9 (25.0%) |  |
|  | Twice | 2 (5.1%) | 2 (5.6%) |  |
|  |  | 3 times | 1 (2.6%) | 0 (0.0%) |

ART; assisted reproductive technology, IUI; intrauterine inseminationSupporting Table 2. Causes of infertility

|  |  | All eligible patients  (Safety analysis set) | Efficacy analysis set |
| --- | --- | --- | --- |
| ^†^ |  | n = 39 | n = 36 |
| Ovulation factors | Ovarian failure | 1 | 1 |
|  | Unknown | 1 | 1 |
| Tubal factors | Tubal obstruction | 6 | 5 |
|  | Tubal adhesion | 1 | 1 |
|  | Chlamydial infection | 2 | 2 |
|  | Unknown | 1 | 1 |
| Uterine factors | Adenomyosis uteri | 4 | 4 |
|  | Endometrial polyp | 6 | 6 |
|  | Uterine myoma | 13 | 12 |
|  | Uterine anomaly | 3 | 2 |
|  | Asherman’s syndrome | 7 | 6 |
|  | Endometrial thinning | 5 | 4 |
|  | Cervical cancer (surgery) | 1 | 1 |
|  | Uterine cancer (conservative therapy) | 1 | 1 |
|  | Cervical cancer (chemotherapy) | 1 | 1 |
|  | Cesarean scar syndrome | 1 | 1 |
|  | Chronic endometriosis | 1 | 1 |
| Endometriosis |  | 6 | 5 |
| Unexplained |  | 19 | 18 |
| male factors |  | 3 | 3 |

^†^If the same patient were suspected of more than one cause of infertility, all factors were counted.

Supporting Table 3. Previous surgeries

|  |  |  |  | All eligible patients (Safety analysis set) | Efficacy analysis set |
| --- | --- | --- | --- | --- | --- |
|  |  |  |  | n = 39 | n = 36 |
| n (%) |  |  |  | 33 (84.6%) | 30 (83.3%) |
| Surgeries^†^ | Hysteroscopic surgery (for polyps and myomas) | | | 14 | 13 |
|  | Adnexal surgery (laparotomy or laparoscopy/including ovarian and fallopian tube surgery) | | | 13 | 10 |
|  | D&C | | | 12 | 10 |
|  | Myomectomy (laparoscopy or laparotomy) | | | 10 | 10 |
|  | Total curettage | | | 9 | 9 |
|  | Hysteroscopic adhesiolysis for Asherman’s syndrome | | | 8 | 7 |
|  | Adenomyomectomy | | | 3 | 3 |
|  | Radical trachelectomy | | | 2 | 2 |
|  | Conization | | | 2 | 2 |
|  | Uterine artery embolization | | | 2 | 2 |
|  | Others | | | 4 | 4 |

^†^If the same patient underwent more than one surgery, all surgeries were counted.
